# Improving accuracy of estimating glomerular filtration rate using artificial neural network: model development and validation

**DOI:** 10.1186/s12967-020-02287-y

**Published:** 2020-03-10

**Authors:** Ningshan Li, Hui Huang, Han-Zhu Qian, Peijia Liu, Hui Lu, Xun Liu

**Affiliations:** 1 SJTU-Yale Joint Center for Biostatistics and Data Science, Department of Bioinformatics and Biostatistics, School of Life Science and Biotechnology, Shanghai Jiao Tong University (SJTU), Room 4-225, Life Science Building, 800 Dongchuan Road, Shanghai, China; 2 Cardiovascular Department, The Eighth Affiliated Hospital, Sun Yat-sen University, Shenzhen, China; 3 Department of Biostatistics, Yale University School of Public Health, New Haven, CT USA; 4 Clinical data center of the Third Affiliated Hospital of Sun Yat sen University, Guangdong, China; 5 Department of Nephrology, The Third Affiliated Hospital of Sun Yat-sen University, Guangdong, China; 6 MoE Key Lab of Artificial Intelligence, AI Institute, Shanghai Jiao Tong University, Shanghai, China; 7 Shanghai Engineering Research Center for Big Data in Pediatric Precision Medicine, Shanghai, China

**Keywords:** Chronic kidney disease, Glomerular filtration rate, Estimating equation, Artificial neural network

## Abstract

**Background:**

The performance of previously published glomerular filtration rate (GFR) estimation equations degrades when directly used in Chinese population. We incorporated more independent variables and using complicated non-linear modeling technology (artificial neural network, ANN) to develop a more accurate GFR estimation model for Chinese population.

**Methods:**

The enrolled participants came from the Third Affiliated Hospital of Sun Yat-sen University, China from Jan 2012 to Jun 2016. Participants with age < 18, unstable kidney function, taking trimethoprim or cimetidine, or receiving dialysis were excluded. Among the finally enrolled 1952 participants, 1075 participants (55.07%) from Jan 2012 to Dec 2014 were assigned as the development data whereas 877 participants (44.93%) from Jan 2015 to Jun 2016 as the internal validation data. We in total developed 3 GFR estimation models: a 4-variable revised CKD-EPI (chronic kidney disease epidemiology collaboration) equation (standardized serum creatinine and cystatin C, age and gender), a 9-variable revised CKD-EPI equation (additional auxiliary variables: body mass index, blood urea nitrogen, albumin, uric acid and hemoglobin), and a 9-variable ANN model.

**Results:**

Compared with the 4-variable equation, the 9-variable equation could not achieve superior performance in the internal validation data (mean of difference: 5.00 [3.82, 6.54] vs 4.67 [3.55, 5.90], P = 0.5; interquartile range (IQR) of difference: 18.91 [17.43, 20.48] vs 20.11 [18.46, 21.80], P = 0.05; P30: 76.6% [73.7%, 79.5%] vs 75.8% [72.9%, 78.6%], P = 0.4), but the 9-variable ANN model significantly improve bias and P30 accuracy (mean of difference: 2.77 [1.82, 4.10], P = 0.007; IQR: 19.33 [17.77, 21.17], P = 0.3; P30: 80.0% [77.4%, 82.7%], P < 0.001).

**Conclusions:**

It is suggested that using complicated non-linear models like ANN could fully utilize the predictive ability of the independent variables, and then finally achieve a superior GFR estimation model.

## Background

Glomerular flirtation rate (GFR) has been well recognized as the best overall indicator of kidney function, which is widely used in diagnosis, treatment and prognosis of chronic kidney disease (CKD) [1]. GFR can be measured by renal or serum clearance of exogenous filtration markers such as inulin and iohexol, but the so-called measured GFR (mGFR) values are cumbersome and costly to be derived in clinical routine. Therefore, investigators have developed widely used GFR estimation equations using established filtration markers (e.g., serum creatinine and cystatin C) in association with demographical variables (e.g., age, gender and race) [2–7]. The global organization kidney disease: improving global outcomes (KDIGO) has recommended to use estimated GFR (eGFR) as the initial test in clinical practice and epidemiological survey [8]. By 2017, many countries have been reporting eGFR with serum creatinine measurement [9].

The most accepted eGFR equations are modification of diet in renal disease (MDRD) [5] and chronic kidney disease epidemiology collaboration (CKD-EPI) equations [7], which can provide acceptable GFR estimates for the North American population. However, these eGFR estimations may not perform well among Chinese population, as these equations were not developed based on Chinese population [10]. Therefore, studies have been conducted to develop accurate equations for Chinese or Asian population [11]. However, most of these studies focus on either establishing an ethnic factor [10] or developing a new equation just using traditional regression method.

In the development of GFR estimation equations, the standard procedures are using natural logarithm transformation of mGFR and filtration markers, then using ordinary least square linear or piecewise linear regression. This simple linearity might not explain the complicated relationship among kidney function, GFR and filtration markers [1, 12]. Moreover, the potential predictive power of auxiliary variables (demographical variables and other laboratory test variables) was not sufficiently utilized, as no interaction terms were incorporated into the equations. Studies have shown that using complicated non-linear modeling technology may improve the performance of GFR estimation [13–16]. Therefore, we used artificial neural network (ANN), a powerful and common methodology in machine learning, to develop a more accurate eGFR model for Chinese population, and validated this model and compared its performance with standard regression equation models.

## Methods

### Study design and study participants

Patients diagnosed with CKD in the Third Affiliated Hospital of Sun Yat-sen University during January 2012 to June 2016 were recruited consecutively into this study. Participates were excluded for any of the following reasons: (1) age < 18 years; (2) having acute kidney function deterioration, skeletal muscle atrophy, edema, pleural effusion or ascites, heart failure, malnutrition, amputation, or ketoacidosis; (3) taking trimethoprim or cimetidine; or (4) receiving dialysis at the time of study.

The institutional review board at the Third Affiliated Hospital of Sun Yat-sen University approved this study. A written informed consent was obtained from all participants.

### Laboratory measurements

GFR was measured by ^99m^Tc-DTPA renal dynamic imaging, which had been recalibrated to a dual plasma sample ^99m^Tc-DTPA GFR. Renal dynamic imaging was obtained with a Millennium TMMPR SPECT using the General Electric Medical System (Discovery VH, GE Healthcare). Serum samples from each participant were collected on the same day of performing GFR measurement and assayed on a Hitachi 7180 auto-analyzer (Hitachi reagents from Roche Diagnostics) in a single laboratory at the Department of Laboratory in the Third Affiliated Hospital of Sun Yet-sun University. Creatinine was measured by an enzymatic method and then recalibrated to the isotope dilution mass spectrometry reference method [17, 18]. We also recalibrated serum cystatin C to the standard reference material (ERM-DA471) [19]. The laboratory test variables were extracted from the analysis report and recorded manually.

### Development of revised CKD-EPI equations and ANN model

The revised equations were derived using the same method for developing the CKD-EPI equation by Inker and colleagues [7]. We first developed an equation for GFR estimation using a combination of conventional 4 variables including *age*, *sex*, *serum creatinine* (*Scr*) and *serum cystatin C* (*Scys*), then we further developed a 9-variable equation by incorporating 5 more auxiliary variables including *body mass index* (*BMI*), *blood urea nitrogen* (*BUN*), *albumin* (*ALB*), *uric acid* (*UA*) and *hemoglobin* (*HGB*). For both equations the dependent variable was mGFR. mGFR and independent variables Scr, Scys, and BUN were log-transformed, so the correlation between mGFR and the independent variables became nearly linear. We developed the equations with 4- and 9-variable which fit the piecewise linear splines with a knot of both Scr and Scys by using *splines* Package in R software (version 3.5.0, R Development Core Team). The method for determining the knot of spline of Scr and Scys was described in Additional file 1.

We also developed an ANN model with the same 9 independent variables for GFR estimation. Prior to ANN development, we performed data cleaning and pre-processing on the development data, including outlier deleting and variable normalization. We used only 1 hidden layer with 4 neurons, and the activation functions in all hidden neurons were set as Leaky ReLU (alpha = 0.1) [20]. The ANN was trained by Stochastic gradient descent (SGD) optimizer, and the whole development of ANN was implemented under Keras framework in Python (version 3.6.6, Python Software Foundation). The detailed ANN model development was described in Additional file 2.

### Model evaluation and statistical analysis

The performance indicators of GFR estimation include bias, precision and accuracy Bias and precision were defined as the median and the interquartile range (IQR) of the difference of eGFR minus mGFR, respectively. Accuracy was assessed as P30 (percentage of eGFR within ± 30% of mGFR). Besides the model evaluation on overall cohort, we also performed the identical evaluation procedures on subgroups divided by mGFR. Data from patients from Jan 2015 to Jun 2016 were used for internal validation on the performance of the derived models. We also performed a sensitivity analysis by developing and internally validating the 3 GFR estimation models based on random split datasets.

Complete-case analysis was used to handle the missing data. The 95% confidence intervals were calculated with bootstrap methods (2000 bootstraps) [21–23]. Wilcoxon signed rank test was used to compare the bias between models, whereas Permutation test for comparison of precision, and McNemar test for comparison of P30. All statistical analysis was performed using MATLAB software (version 2018b, MathWorks).

## Results

### Characteristics of participants

**Fig. 1:**
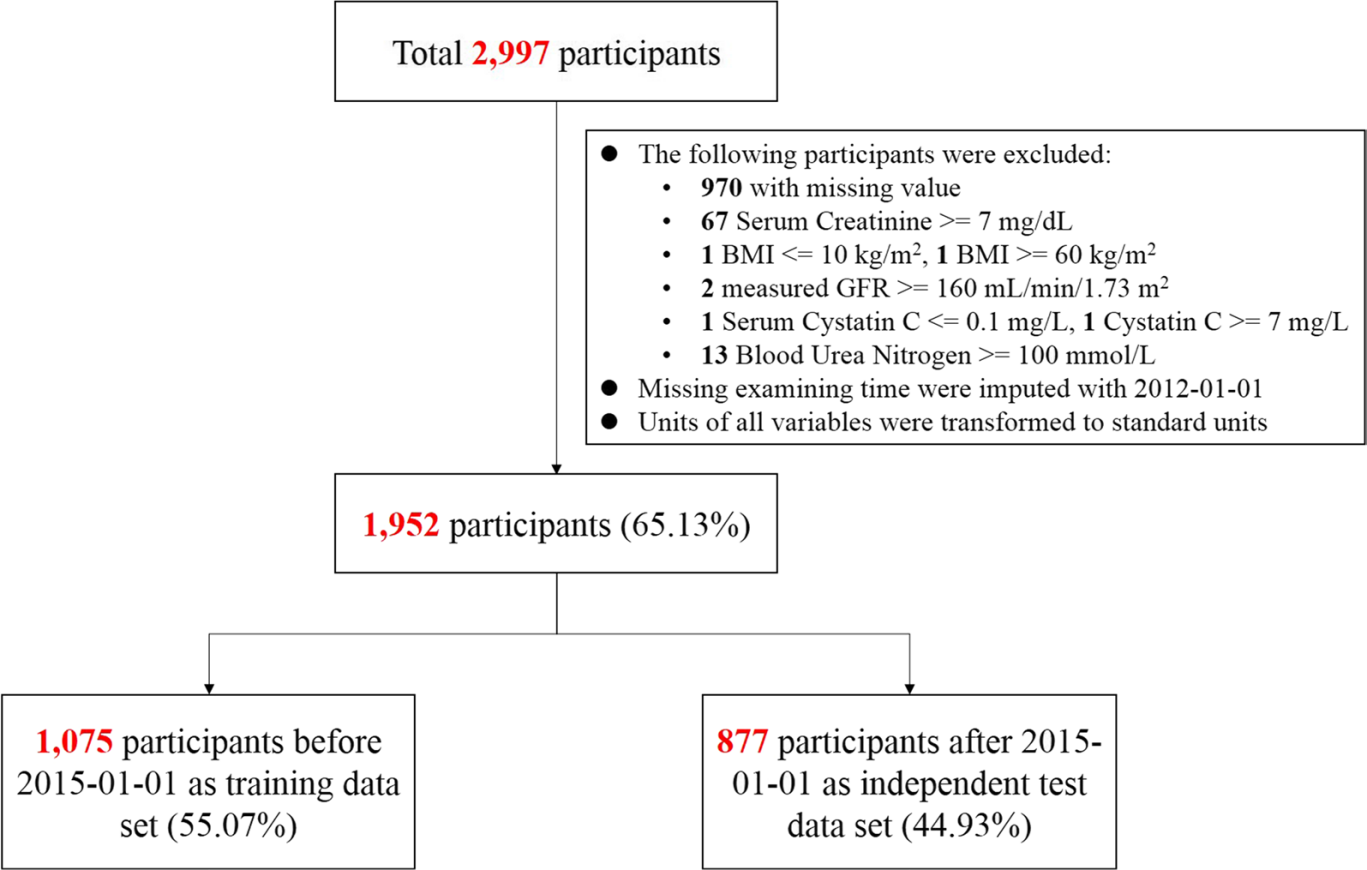
Flowchart of study design. *GFR* glomerular filtration rate, *AAK* African American Study of Kidney Disease and Hypertension, *BMI* body mass index

Among the initially enrolled 2997 CKD patients during 2012 and 2016, 970 with incomplete data and 75 with irregular recordings were excluded (details are available in Fig. 1). Finally, 1952 participants were included in the model development or validation, including 1075 (55.1%) participants from Jan 2012 to Dec 2014 assigned into the development dataset to derive the revised equations and ANN, whereas 877 (44.9%) from Jan 2015 to Jun 2016 assigned as the internal validation dataset to independently evaluate the performance of the derived models.

Table 1 summarizes the characteristics of both development and internal validation datasets. For the development dataset, 57.3% were male; mean age was 55.6 years (standard deviation [SD] 14.5); mean mGFR was 71.0 (SD, 27.4) mL/min/1.73 m^2^, serum creatinine 1.5 (SD, 1.3) mg/dL, and serum cystatin C 1.5 (SD, 0.9) mg/L. For internal validation dataset, 59.0% were male; mean age was 57.4 (SD, 13.4) years; mGFR was 68.8 (27.1) mL/min/1.73 m^2^, serum creatinine 1.3 (SD, 0.9) mg/dL, and serum cystatin C 1.3 (SD, 0.7) mg/L. There were few participants with mGFR less than 30 mL/min/1.73 m^2^ in both development and internal validation dataset (6.5% and 6.3% respectively).

**Table 1:** Characteristics of participants in the development and internal validation datasets

Variable	Development dataset (N = 1075)	Internal validation dataset (N = 877)	P value
Age (year)	55.6 ± 14.5	57.4 ± 13.4	0.008
Male sex, N (%)	616 (57.3)	517 (59.0)	0.5
Body mass index (kg/m^2^)	24.0 ± 3.6	24.5 ± 3.8	0.005
Serum creatinine (mg/dL)*	1.5 ± 1.3	1.3 ± 0.9	< 0.001
Serum cystatin C (mg/L)	1.5 ± 0.9	1.3 ± 0.7	< 0.001
Blood urea nitrogen (mg/dL)	20.9 ± 13.5	21.5 ± 11.7	0.3
Albumin (g/dL)	3.9 ± 0.5	3.9 ± 0.5	0.3
Uric acid (mg/dL)	6.7 ± 2.1	7.1 ± 2.2	< 0.001
Hemoglobin (g/L)	12.3 ± 2.3	12.7 ± 2.1	< 0.001
Measured GFR (mL/min/1.73 m^2^)	71.0 ± 27.4	68.8 ± 27.1	0.08
Measured GFR N(%)			0.04
< 30 mL/min/1.73 m^2^	70 (6.5)	55 (6.3)	
30–59 mL/min/1.73 m^2^	310 (28.8)	304 (34.7)	
60–89 mL/min/1.73 m^2^	420 (39.1)	324 (36.9)	
≥ 90 mL/min/1.73 m^2^	275 (25.6)	194 (22.1)	

Values for continuous variables were reported as mean ± standard deviation, and values for categorical variables as number (percentage). Conversion factor for units: serum creatinine in mg/dL to mmol/L × 88.4

*GFR* glomerular filtration rate

### Formulation of revised CKD-EPI equations and ANN model

The knots of serum creatinine for female and male participants were 0.7 and 0.9 mg/dL, respectively, whereas the knots of serum cystatin C were both 0.9 mg/L. The formulations of revised 4-variable and 9-variable CKD-EPI equations were shown in Table 2. Additional file 3 shows how to implement the 9-variable ANN model.

**Table 2:** The formulations for the revised 4-variable and 9-variable CKD-EPI equations

Sex	Serum creatinine, mg/dL	Serum cystatin C, mg/L	4-variable equation	9-variable equation
Female	≤ 0.7	≤ 0.9	$$122 \times \left( {{\raise0.7ex\hbox{${Scr}$} \!\mathord{\left/ {\vphantom {{Scr} {0.7}}}\right.\kern-0pt} \!\lower0.7ex\hbox{${0.7}$}}} \right)^{ - 0.236} \times \left( {{\raise0.7ex\hbox{${Scys}$} \!\mathord{\left/ {\vphantom {{Scys} {0.9}}}\right.\kern-0pt} \!\lower0.7ex\hbox{${0.9}$}}} \right)^{ - 0.078} \times 0.995^{Age}$$	$$154 \times \left( {{\raise0.7ex\hbox{${Scr}$} \!\mathord{\left/ {\vphantom {{Scr} {0.7}}}\right.\kern-0pt} \!\lower0.7ex\hbox{${0.7}$}}} \right)^{ - 0.262} \times \left( {{\raise0.7ex\hbox{${Scys}$} \!\mathord{\left/ {\vphantom {{Scys} {0.9}}}\right.\kern-0pt} \!\lower0.7ex\hbox{${0.9}$}}} \right)^{ - 0.045} \times 0.996^{Age} \times 0.985^{BMI} \times BUN^{0.006} \times 0.985^{ALB} \times 0.997^{UA} \times 1.012^{HGB}$$
		> 0.9	$$122 \times \left( {{\raise0.7ex\hbox{${Scr}$} \!\mathord{\left/ {\vphantom {{Scr} {0.7}}}\right.\kern-0pt} \!\lower0.7ex\hbox{${0.7}$}}} \right)^{ - 0.236} \times \left( {{\raise0.7ex\hbox{${Scys}$} \!\mathord{\left/ {\vphantom {{Scys} {0.9}}}\right.\kern-0pt} \!\lower0.7ex\hbox{${0.9}$}}} \right)^{ - 0.381} \times 0.995^{Age}$$	$$154 \times \left( {{\raise0.7ex\hbox{${Scr}$} \!\mathord{\left/ {\vphantom {{Scr} {0.7}}}\right.\kern-0pt} \!\lower0.7ex\hbox{${0.7}$}}} \right)^{ - 0.262} \times \left( {{\raise0.7ex\hbox{${Scys}$} \!\mathord{\left/ {\vphantom {{Scys} {0.9}}}\right.\kern-0pt} \!\lower0.7ex\hbox{${0.9}$}}} \right)^{ - 0.374} \times 0.996^{Age} \times 0.985^{BMI} \times BUN^{0.006} \times 0.985^{ALB} \times 0.997^{UA} \times 1.012^{HGB}$$
Female	> 0.7	≤ 0.9	$$122 \times \left( {{\raise0.7ex\hbox{${Scr}$} \!\mathord{\left/ {\vphantom {{Scr} {0.7}}}\right.\kern-0pt} \!\lower0.7ex\hbox{${0.7}$}}} \right)^{ - 0.330} \times \left( {{\raise0.7ex\hbox{${Scys}$} \!\mathord{\left/ {\vphantom {{Scys} {0.9}}}\right.\kern-0pt} \!\lower0.7ex\hbox{${0.9}$}}} \right)^{ - 0.078} \times 0.995^{Age}$$	$$154 \times \left( {{\raise0.7ex\hbox{${Scr}$} \!\mathord{\left/ {\vphantom {{Scr} {0.7}}}\right.\kern-0pt} \!\lower0.7ex\hbox{${0.7}$}}} \right)^{ - 0.320} \times \left( {{\raise0.7ex\hbox{${Scys}$} \!\mathord{\left/ {\vphantom {{Scys} {0.9}}}\right.\kern-0pt} \!\lower0.7ex\hbox{${0.9}$}}} \right)^{ - 0.045} \times 0.996^{Age} \times 0.985^{BMI} \times BUN^{0.006} \times 0.985^{ALB} \times 0.997^{UA} \times 1.012^{HGB}$$
		> 0.9	$$122 \times \left( {{\raise0.7ex\hbox{${Scr}$} \!\mathord{\left/ {\vphantom {{Scr} {0.7}}}\right.\kern-0pt} \!\lower0.7ex\hbox{${0.7}$}}} \right)^{ - 0.330} \times \left( {{\raise0.7ex\hbox{${Scys}$} \!\mathord{\left/ {\vphantom {{Scys} {0.9}}}\right.\kern-0pt} \!\lower0.7ex\hbox{${0.9}$}}} \right)^{ - 0.381} \times 0.995^{Age}$$	$$154 \times \left( {{\raise0.7ex\hbox{${Scr}$} \!\mathord{\left/ {\vphantom {{Scr} {0.7}}}\right.\kern-0pt} \!\lower0.7ex\hbox{${0.7}$}}} \right)^{ - 0.320} \times \left( {{\raise0.7ex\hbox{${Scys}$} \!\mathord{\left/ {\vphantom {{Scys} {0.9}}}\right.\kern-0pt} \!\lower0.7ex\hbox{${0.9}$}}} \right)^{ - 0.374} \times 0.996^{Age} \times 0.985^{BMI} \times BUN^{0.006} \times 0.985^{ALB} \times 0.997^{UA} \times 1.012^{HGB}$$
Male	≤ 0.9	≤ 0.9	$$107 \times \left( {{\raise0.7ex\hbox{${Scr}$} \!\mathord{\left/ {\vphantom {{Scr} {0.9}}}\right.\kern-0pt} \!\lower0.7ex\hbox{${0.9}$}}} \right)^{ - 0.292} \times \left( {{\raise0.7ex\hbox{${Scys}$} \!\mathord{\left/ {\vphantom {{Scys} {0.9}}}\right.\kern-0pt} \!\lower0.7ex\hbox{${0.9}$}}} \right)^{ - 0.045} \times 0.996^{Age}$$	$$103 \times \left( {{\raise0.7ex\hbox{${Scr}$} \!\mathord{\left/ {\vphantom {{Scr} {0.9}}}\right.\kern-0pt} \!\lower0.7ex\hbox{${0.9}$}}} \right)^{ - 0.347} \times \left( {{\raise0.7ex\hbox{${Scys}$} \!\mathord{\left/ {\vphantom {{Scys} {0.9}}}\right.\kern-0pt} \!\lower0.7ex\hbox{${0.9}$}}} \right)^{ - 0.099} \times 0.996^{Age} \times 0.982^{BMI} \times BUN^{0.125} \times 0.949^{ALB} \times 0.996^{UA} \times 1.027^{HGB}$$
		> 0.9	$$107 \times \left( {{\raise0.7ex\hbox{${Scr}$} \!\mathord{\left/ {\vphantom {{Scr} {0.9}}}\right.\kern-0pt} \!\lower0.7ex\hbox{${0.9}$}}} \right)^{ - 0.292} \times \left( {{\raise0.7ex\hbox{${Scys}$} \!\mathord{\left/ {\vphantom {{Scys} {0.9}}}\right.\kern-0pt} \!\lower0.7ex\hbox{${0.9}$}}} \right)^{ - 0.397} \times 0.996^{Age}$$	$$103 \times \left( {{\raise0.7ex\hbox{${Scr}$} \!\mathord{\left/ {\vphantom {{Scr} {0.9}}}\right.\kern-0pt} \!\lower0.7ex\hbox{${0.9}$}}} \right)^{ - 0.347} \times \left( {{\raise0.7ex\hbox{${Scys}$} \!\mathord{\left/ {\vphantom {{Scys} {0.9}}}\right.\kern-0pt} \!\lower0.7ex\hbox{${0.9}$}}} \right)^{ - 0.405} \times 0.996^{Age} \times 0.982^{BMI} \times BUN^{0.125} \times 0.949^{ALB} \times 0.996^{UA} \times 1.027^{HGB}$$
Male	> 0.9	≤ 0.9	$$107 \times \left( {{\raise0.7ex\hbox{${Scr}$} \!\mathord{\left/ {\vphantom {{Scr} {0.9}}}\right.\kern-0pt} \!\lower0.7ex\hbox{${0.9}$}}} \right)^{ - 0.358} \times \left( {{\raise0.7ex\hbox{${Scys}$} \!\mathord{\left/ {\vphantom {{Scys} {0.9}}}\right.\kern-0pt} \!\lower0.7ex\hbox{${0.9}$}}} \right)^{ - 0.045} \times 0.996^{Age}$$	$$103 \times \left( {{\raise0.7ex\hbox{${Scr}$} \!\mathord{\left/ {\vphantom {{Scr} {0.9}}}\right.\kern-0pt} \!\lower0.7ex\hbox{${0.9}$}}} \right)^{ - 0.391} \times \left( {{\raise0.7ex\hbox{${Scys}$} \!\mathord{\left/ {\vphantom {{Scys} {0.9}}}\right.\kern-0pt} \!\lower0.7ex\hbox{${0.9}$}}} \right)^{ - 0.099} \times 0.996^{Age} \times 0.982^{BMI} \times BUN^{0.125} \times 0.949^{ALB} \times 0.996^{UA} \times 1.027^{HGB}$$
		> 0.9	$$107 \times \left( {{\raise0.7ex\hbox{${Scr}$} \!\mathord{\left/ {\vphantom {{Scr} {0.9}}}\right.\kern-0pt} \!\lower0.7ex\hbox{${0.9}$}}} \right)^{ - 0.358} \times \left( {{\raise0.7ex\hbox{${Scys}$} \!\mathord{\left/ {\vphantom {{Scys} {0.9}}}\right.\kern-0pt} \!\lower0.7ex\hbox{${0.9}$}}} \right)^{ - 0.397} \times 0.996^{Age}$$	$$103 \times \left( {{\raise0.7ex\hbox{${Scr}$} \!\mathord{\left/ {\vphantom {{Scr} {0.9}}}\right.\kern-0pt} \!\lower0.7ex\hbox{${0.9}$}}} \right)^{ - 0.391} \times \left( {{\raise0.7ex\hbox{${Scys}$} \!\mathord{\left/ {\vphantom {{Scys} {0.9}}}\right.\kern-0pt} \!\lower0.7ex\hbox{${0.9}$}}} \right)^{ - 0.405} \times 0.996^{Age} \times 0.982^{BMI} \times BUN^{0.125} \times 0.949^{ALB} \times 0.996^{UA} \times 1.027^{HGB}$$

*CKD-EPI* chronic kidney disease epidemiology collaboration, *Scr* serum creatinine, *Scys* serum cystatin C, *BMI* body mass index, *BUN* blood urea nitrogen, *ALB* albumin, *UA* uric acid, *HGB* hemoglobin

### Performance of models in the internal validation dataset

The performance of three derived models was summarized in Table 3. The bias (median difference) between mGFR and eGFR of the revised 4-variable CKD-EPI equation is 4.67 [95% CI 3.55–5.90], the precision 20.11 [18.46–21.80] mL/min/1.73 m^2^, and the accuracy (or P30) 75.8% [72.9–78.6%].

**Table 3:** Comparison of the performance of revised 4-variable and 9-variable CKD-EPI equations and 9-variable ANN model: internal validation

	Overall	Measured GFR < 60 mL/min/1.73 m^2^	Measured GFR ≥ 60 mL/min/1.73 m^2^
Bias—median difference (95% CI)			
4-variable CKD-EPI equation	4.67 (3.55 to 5.90)	11.04 (9.47 to 12.32)	0.03 (− 1.73 to 1.18)
9-variable CKD-EPI equation	5.00 (3.82 to 6.54) P = 0.5	10.95 (9.08 to 12.60) P = 0.8	− 0.10 (− 1.54 to 2.09) P = 0.3
9-variable ANN	2.77 (1.82 to 4.10) P = 0.007	10.54 (8.40 to 11.78) P = 0.2	− 2.91 (− 4.60 to − 1.32) P = 0.01
Precision – IQR of the difference (95% CI)			
4-variable CKD-EPI equation	20.11 (18.46 to 21.80)	15.90 (13.90 to 17.87)	21.08 (19.34 to 23.80)
9-variable CKD-EPI equation	18.91 (17.43 to 20.48) P = 0.05	16.73 (14.67 to 19.05) P = 0.3	21.01 (18.69 to 23.78) P = 0.9
9-variable ANN	19.33 (17.77 to 21.17) P = 0.3	16.03 (14.15 to 17.72) P = 0.9	20.80 (19.19 to 22.84) P = 0.7
Accuracy—P30, % (95% CI)			
4-variable CKD-EPI equation	75.8 (72.9 to 78.6)	53.2 (47.9 to 58.2)	91.5 (88.8 to 93.6)
9-variable CKD-EPI equation	76.6 (73.7 to 79.5) P = 0.4	54.6 (49.6 to 59.7) P = 0.4	91.9 (89.6 to 94.0) P = 0.7
9-variable ANN	80.0 (77.4 to 82.7) P < 0.001	59.9 (54.6 to 64.9) P < 0.001	94.0 (91.5 to 95.9) P = 0.01

*GFR* glomerular filtration rate, *CKD-EPI* chronic kidney disease epidemiology collaboration, *ANN* artificial neural network, *IQR* interquartile range, *CI* confidence interval

Compared with the revised 4-variable CKD-EPI equation, the 9-variable equation has similar bias (5.00 [3.82–6.54] mL/min/1.73 m^2^, P = 0.5) and P30 (76.6% [73.7–79.5%], P = 0.4), and a slightly better precision (18.91 [17.43–20.48] mL/min/1.73 m^2^, P = 0.05).

The bias of 9-variable ANN model is 2.77 [1.82–4.10] mL/min/1.73 m^2^, which is much smaller than that of 4-variable revised CKD-EPI equation (P = 0.007). The P30 of ANN model is 80.0% [77.4–82.7%], significantly higher than the two equation (P < 0.001). However, it is similar in precision between ANN and the 4-variable equation (P = 0.3, see Table 3).

The model performance in subgroups by mGFR was similar with the overall performance. In both subgroups of mGFR < 60 mL/min/1.73 m^2^ and mGFR ≥ 60 mL/min/1.73 m^2^, the 9-variable ANN model consistently achieved superior P30 than the two revised equations. However, in subgroup of mGFR ≥ 90 mL/min/1.73 m^2^ the ANN model tended to be more biased (median of difference − 2.91 [− 4.60 to − 1.32] mL/min/1.73 m^2^) (Table 3).

The sensitivity analysis based on random split datasets showed that the 9-variable ANN model has significantly superior P30 and precision and similar bias compared with the 4-variable CKD-EPI equation (see Additional file 4).

## Discussion

Accurate evaluation of GFR is important for assessing the severity of CKD, predicting prognosis and deciding proper therapeutic interventions. Since publication of Cockcroft-Gault (CG) Equation in 1976 [2], many studies have been conducted to derive actionable models to estimate GFR. The major barrier of accurately estimate individual’s GFR is non-GFR determinants of filtration markers [1, 12, 24, 25], which degrade the ideal linear correlation between GFR and filtration markers. Under the consideration of cost and convenience, such unmeasured non-GFR determinants are unable to be incorporated into the GFR estimation models, instead auxiliary variables (demographical variables and other laboratory test variables) are used as surrogates. The frequently used demographical variables are age, gender and race, whereas the frequently used other laboratory test variables are blood urea nitrogen and albumin.

However, other laboratory test variables in the linear equations seem to have limited predictive ability to estimate GFR compared with filtration markers and demographical variables. The 6-variable MDRD equation has two additional variables Serum urea nitrogen and Albumin than the simplified 4-variable MDRD equation, but the performance of the two equations are nearly the same [3–5]. In the development of CKD-EPI equation in 2012, no other laboratory test variables or interaction terms are incorporated into the final equation as their predictive ability are not statistically significant during variable selection [7].

In our study, we developed two revised CKD-EPI equations. One equation incorporated 4 variables: standardized serum creatinine and cystatin C, age and gender, which are the standard variable combination during developing the GFR estimation model. We further incorporated more auxiliary variables as in theory it is beneficial using more independent variables when developing prediction models. Besides blood urea nitrogen and albumin, we also incorporated body mass index, uric acid and hemoglobin, and finally developed a 9-variable revised CKD-EPI equation. However, the two revised equations turned out to have similar performance of GFR estimation. The reason behind this phenomenon is the simple linear regression cannot sufficiently utilize the potential predictive power of these auxiliary variables. When we used the same 9 variables to develop a ANN model, compared with the revised 4-variable CKD-EPI equation, the 9-variable ANN model significantly reduce bias and improve P30 accuracy.

The mathematical theory of ANN is the universal approximation theorem [26, 27], which means that ANN is able to approximate any continuous even uncontinuous functions. When the network size of ANN increases, the capacity of ANN will become more powerful. Furthermore, ANN doesn’t require any assumptions about distribution of variables and can handle with the multi-collinearity among independent variables [28]. Therefore, ANN can capture not only the complicated correlations between GFR and independent variables, but also any interactions between independent variables, so it can make GFR estimations based on these sophisticated relationships.

In future GFR estimation studies, it is a major trend to incorporated more variables into GFR estimation models, such as potential novel filtration markers β-Trace Protein (BTP) and β2-Microglobulin (B2M) [29–31], and even biomarkers from proteomics and metabolomics [30–32]. Our study suggests that it is beneficial to use complicated models to fully utilize the predictive ability of these variables to achieve a good performance of GFR estimation.

There are limitations in our study. First, all study participants were from one medical center in China, and most are CKD patients. The generalizability of the study may be limited to CKD patients, and the performance of the developed ANN still requires extra validation on diverse populations. Second, the gold standard mGFR was measured by ^99m^Tc-DTPA renal dynamic imaging, and then recalibrated to a dual plasma sample ^99m^Tc-DTPA GFR. It is widely accepted that using iohexol or iothalamate will achieve a more accurate mGFR compared with ^99m^Tc-DTPA [33]. Third, the sizes of development dataset as well as internal validation dataset are relatively small, especially there were few participants with mGFR ≤ 30 mL/min/1.73 m^2^. Fourth, although ANN model is superior in the accuracy, it is difficult to interpret, and the relationship and interaction between independent variables are still unknown.

## Conclusions

In conclusion, we introduced up to 9 variables into GFR estimation and developed revised CKD-EPI 4-variable and 9-variable equations, and a 9-variable ANN model, respectively. Compared with the 4-variable equation, the 9-variable equation could not achieve superior performance, but the 9-variable ANN model significantly reduces bias and improve P30 accuracy. It is suggested that using complicated non-linear models like ANN could fully utilize the predictive ability of the additional auxiliary variables. However, the proposed ANN model still requires extra and careful validation in more diverse cohort data.

## Supplementary information

**Additional file 1.** Determining the knot of spline of serum creatinine and serum cystatin C.

**Additional file 2.** Development of the ANN model.

**Additional file 3.** An Excel file to implement the 9-variable ANN model.

**Additional file 4.** Sensitivity analysis of data splitting method.

## Data Availability

The datasets used and/or analyzed during the current study are available from the corresponding author on reasonable request.

## Abbreviations

CKD: Chronic kidney disease
GFR: Glomerular filtration rate
mGFR: Measured glomerular filtration rate
eGFR: Estimated glomerular filtration rate
KDIGO: Kidney disease: improving global outcomes
CG: Cockcroft-Gault
MDRD: Modification of diet in renal disease
CKD-EPI: Chronic kidney disease epidemiology collaboration
ANN: Artificial neural network
BMI: Body mass index
Scr: Serum creatinine
Scys: Serum cystatin C
BUN: Blood urea nitrogen
ALB: Albumin
UA: Uric acid
HGB: Hemoglobin
BTP: β-Trace Protein
B2M: β2-Microglobulin
IQR: Interquartile range
SD: Standard deviation
