# Supplementary material for: Improving accuracy of estimating glomerular filtration rate using artificial neural network: model development and validation
Source: J Transl Med. 2020 Mar 10;18:120. doi: 10.1186/s12967-020-02287-y (PMC7063770; doi:10.1186/s12967-020-02287-y)
Supplement: Supplementary file 1 — Additional file 1. Determining the knot of spline of serum creatinine and serum cystatin C. [file 12967_2020_2287_MOESM1_ESM.docx]

# Additional file 1. Determining the knot of spline of Scr and Scys

The CKD-EPI (Chronic Kidney Disease Epidemiology Collaboration) equation was essentially a piecewise linear spline connected by just one knot of both serum creatinine (Scr) and serum cystatin C (Scys). Therefore, we must first determine the optimal knot of Scr and Scys for each cohort grouped by sex. We used grid search to select an optimal knot, and the grid interval was 0.1 mg/dL for Scr and 0.1 mg/L for Scys respectively. For each knot candidate, we repeated 5-fold cross-validation 100 times, and finally the optimal knot was selected based on the average of root of mean squared error (RMSE) over 100 results.

Finally, the knot of serum creatinine for female and male were 0.7 and 0.9 mg/dL respectively, whereas the knot of serum cystatin C was 0.9 mg/L for both female and male. The box plot of RMSE of 5-fold cross-validation was shown in *Additional Figure S1* below.

| 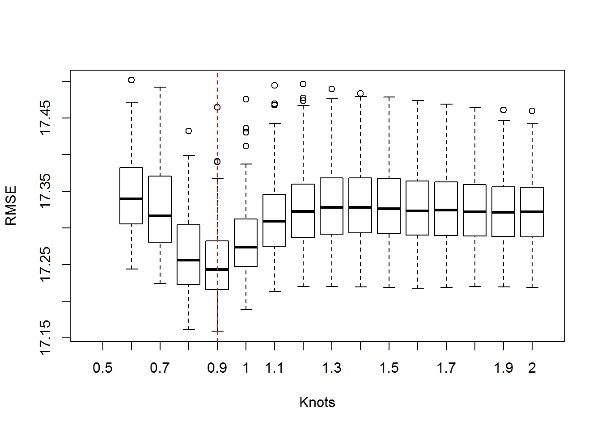  A1. Scr knot for male group | 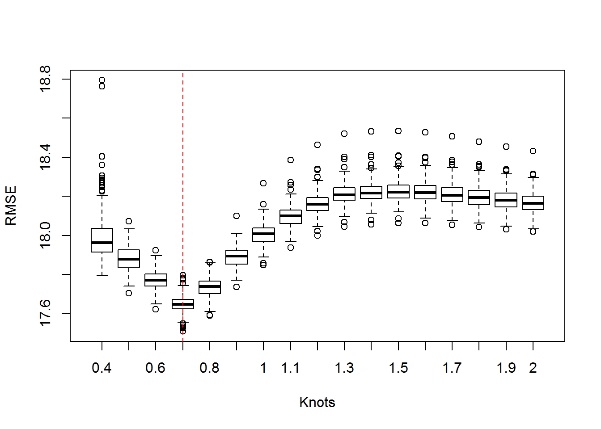  A2. Scr knot for female group |
| --- | --- |
| 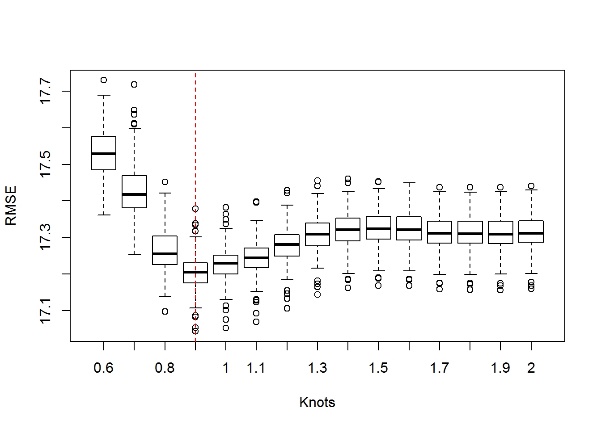  B1. Scys knot for male group | 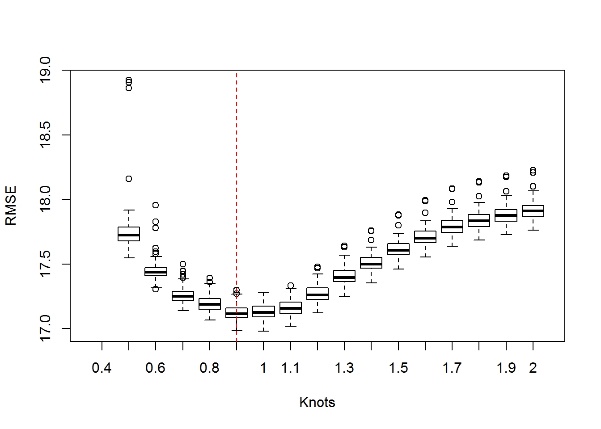  B2. Scys knot for female group |
| **Additional Figure S1**. Box plot of RMSE of 5-fold cross-validation with 100 repetitions for revised CKD-EPI equations. The red vertical dashed line represented the selected knot of Scr and Scys with the lowest average of RMSE over 100 results.  **Abbreviations:** RMSE, root of mean squared error; CKD-EPI, Chronic Kidney Disease Epidemiology Collaboration; Scr, serum creatinine; Scys, serum cystatin C. | |
