# Supplementary material for: Improving accuracy of estimating glomerular filtration rate using artificial neural network: model development and validation
Source: J Transl Med. 2020 Mar 10;18:120. doi: 10.1186/s12967-020-02287-y (PMC7063770; doi:10.1186/s12967-020-02287-y)
Supplement: Supplementary file 2 — Additional file 2. Development of the ANN model. [file 12967_2020_2287_MOESM2_ESM.docx]

# Additional file 2. Development of ANN model

We proposed an artificial neural network (ANN) model with 9 independent variables for glomerular filtration rate (GFR) estimation, and the 9 independent variables included *age*, *sex,* *serum creatinine* (*Scr*), *serum cystatin C* (*Scys*), *body mass index* (*BMI*), *blood urea nitrogen* (*BUN*), *albumin* (*ALB*), *uric acid* (*UA*) and *hemoglobin* (*HGB*).

## Data preprocessing

We first excluded 13 outliers from the pooled labeled data set, as complicated modeling algorithms just like ANN were sensitive to outliers.

To train ANN efficiently, the variables used for modeling were pre-processed before training. The coding of binary variable *sex* was changed from 0, 1 to -1, 1. The continuous variables *age*, *BMI*, *ALB*, *UA*, and *HGB* were linearly normalized to range [-1, 1], while *Scr*, *Scys* and *BUN* were log-transformed, and followed by linearly normalization to range [-1, 1]. The dependent variable measure GFR was only linearly normalized to range [-1, 1] and none natural logarithmic transformed was performed.

## Development of ANN model

The development of the ANN model was implemented under Keras framework in Python (version 3.6.6, Python Software Foundation). The detailed set up of ANN was described below.

- The number of neurons in the input layer was corresponding to the number of independent variables (9), while the number of neurons in the output layer was just 1 corresponding to the dependent variable (measured GFR)
- There was only 1 hidden layer which has 4 neurons.
- The activation functions in all hidden neurons were Leaky ReLU (alpha = 0.1) [1]. The activation function in the neuron of output layer was identity function.
- Batch size for each training iteration was set to 256.
- The total training epoch was set to 200.
- The coefficient of L2 regularization for weights was set to 0.005.
- Biases of each neuron were initialized to 0, while weights between layers were initialized using Glorot uniform method [2].
- Using Stochastic gradient descent (SGD) optimizer. The learning rate was set to 0.05, the momentum coefficient was set to 0.9, none learning rate decay, and using Nesterov momentum.

The estimated GFR was just the output of ANN, and it was so complicated to be formulated as equations, so we instead provided an Excel file (Additional file 3) to implement this ANN model.

# References

1. Maas AL, Hannun AY, Ng AY: **Rectifier nonlinearities improve neural network acoustic models.** In *ICML (International Conference on Machine Learning); Atlanta, Georgia, USA*. 2013: 3.

2. Glorot X, Bengio Y: **Understanding the difficulty of training deep feedforward neural networks.** In *Proceedings of the Thirteenth International Conference on Artificial Intelligence and Statistics* (Yee Whye T, Mike T eds.), vol. 9. pp. 249--256. Proceedings of Machine Learning Research: PMLR; 2010:249--256.
