# Supplementary material for: Improving accuracy of estimating glomerular filtration rate using artificial neural network: model development and validation
Source: J Transl Med. 2020 Mar 10;18:120. doi: 10.1186/s12967-020-02287-y (PMC7063770; doi:10.1186/s12967-020-02287-y)
Supplement: Supplementary file 4 — Additional file 4. Sensitivity analysis of data splitting method. [file 12967_2020_2287_MOESM4_ESM.docx]

# Additional file 4. Sensitivity analysis of data splitting method

We performed sensitivity analysis using random data splitting method, that is, the development and internal validation datasets used for glomerular filtration rate (GFR) estimation models development were derived by randomly splitting the whole dataset rather than splitting the data based on time participant recruitment. The results from both methods are similar.

## Characteristics of Participants

The whole dataset (N = 1,952) was randomly split into a development dataset (N = 976) and an internal validation dataset (N = 976). The characteristics of both development and internal validation datasets was summarized in *Additional Table S2*.

## Determining the knot of spline of Scr and Scys

We used the same methodology to determine the optimal knots in revised CKD-EPI (Chronic Kidney Disease Epidemiology Collaboration) equation. The knot of serum creatinine (Scr) for female and male were 0.7 and 0.6 mg/dL respectively, whereas the knot of serum cystatin C (Scys) was 0.8 mg/L for both female and male. The box plot of root of mean squared error (RMSE) of 5-fold cross-validation was shown in *Additional Figure S2*.

## Formulation of revised CKD-EPI equations

The formulations of revised 4-variable and 9-variable CKD-EPI equations were shown in *Additional Table S3*.

## Performance of models in the internal validation dataset

The performance of 3 derived models was summarized in *Additional Table S4*. The bias (median difference) between measured GFR and estimated GFR of the revised 4-variable CKD-EPI equation is -0.57 [95% CI, -2.00 to 0.41], the precision 20.66 [18.90 to 22.15] mL/min/1.73 m^2^, and the accuracy (or P30) 80.0% [77.5% to 82.6%].

Compared with the revised 4-variable CKD-EPI equation, the 9-variable equation has worse bias (-1.98 [-3.27 to -1.06], P = 0.06), better precision (19.18 [17.75 to 20.93] mL/min/1.73 m^2^, P = 0.01), and slightly better P30 (81.5% [78.9% to 83.8%], P = 0.1).

The bias of 9-variable ANN model is -0.14 [-1.08 to 0.93] mL/min/1.73 m^2^, which is similar with that of 4-variable revised CKD-EPI equation (P = 0.5). The precision and P30 of ANN model is 18.23 [16.96 to 19.55] mL/min/1.73 m^2^ and 83.5% [81.0% to 85.8%] respectively, both significantly better than the 4-variable CKE-EPI equation (both P < 0.001).

**Additional Table S2**. Characteristics of participants in the development and internal validation datasets

| Variable | Development dataset  (N = 976) | Internal validation dataset  (N = 976) | P value |
| --- | --- | --- | --- |
| Age (year) | 56.6 ± 13.9 | 56.2 ± 14.1 | 0.5 |
| Male sex, N (%) | 560 (57.4) | 573 (59.7) | 0.6 |
| Body mass index (kg/m^2^) | 24.2 ± 3.7 | 24.2 ± 3.7 | 0.6 |
| Serum creatinine (mg/dL)* | 1.4 ± 1.1 | 1.4 ± 1.1 | 0.9 |
| Serum cystatin C (mg/L) | 1.4 ± 0.9 | 1.4 ± 0.8 | 0.8 |
| Blood urea nitrogen (mg/dL) | 21.1 ± 12.4 | 21.3 ± 13.1 | 0.7 |
| Albumin (g/dL) | 3.9 ± 0.5 | 3.9 ± 0.5 | 0.2 |
| Uric acid (mg/dL) | 6.9 ± 2.1 | 6.9 ± 2.2 | 0.8 |
| Hemoglobin (g/L) | 12.5 ± 2.2 | 12.5 ± 2.2 | 0.7 |
| Measured GFR (mL/min/1.73 m^2^) | 70.1 ± 27.3 | 70.0 ± 27.3 | 0.9 |
| Measured GFR N (%) |  |  | 0.9 |
| < 30 mL/min/1.73 m^2^ | 66 (6.8) | 59 (6.0) |  |
| 30-59 mL/min/1.73 m^2^ | 303 (31.0) | 311 (31.9) |  |
| 60-89 mL/min/1.73 m^2^ | 371 (38.0) | 373 (38.2) |  |
| ≥ 90 mL/min/1.73 m^2^ | 236 (24.2) | 233 (23.9) |  |

Note: Values for continuous variables were reported as mean ± standard deviation, and values for categorical variables as number (percentage). Conversion factor for units: serum creatinine in mg/dL to mmol/L ×88.4

**Abbreviations**: GFR, glomerular filtration rate.

**Additional Table S3. The formulations for the revised 4-variable and 9-variable CKD-EPI equations**

| Sex | Serum creatinine, mg/dL | Serum cystatin C, mg/L | | 4-variable equation | 9-variable equation |
| --- | --- | --- | --- | --- | --- |
| Female | ≤ 0.7 | ≤ 0.8 | $129{\times(\frac{Scr}{0.7})}^{-0.303}{\times(\frac{Scys}{0.8})}^{-0.159}\times{0.994}^{Age}$ | | $188{\times(\frac{Scr}{0.7})}^{-0.294}{\times(\frac{Scys}{0.8})}^{-0.086}\times{0.995}^{Age}\times{0.981}^{BMI}\times{BUN}^{-0.069}\times{1.004}^{ALB}\times{0.991}^{UA}\times{1.017}^{HGB}$ |
|  |  | > 0.8 | $129{\times(\frac{Scr}{0.7})}^{-0.303}{\times(\frac{Scys}{0.8})}^{-0.358}\times{0.994}^{Age}$ | | $188{\times(\frac{Scr}{0.7})}^{-0.294}{\times(\frac{Scys}{0.8})}^{-0.261}\times{0.995}^{Age}\times{0.981}^{BMI}\times{BUN}^{-0.069}\times{1.004}^{ALB}\times{0.991}^{UA}\times{1.017}^{HGB}$ |
| Female | > 0.7 | ≤ 0.8 | $129{\times(\frac{Scr}{0.7})}^{-0.360}{\times(\frac{Scys}{0.8})}^{-0.159}\times{0.994}^{Age}$ | | $188{\times(\frac{Scr}{0.7})}^{-0.348}{\times(\frac{Scys}{0.8})}^{-0.086}\times{0.995}^{Age}\times{0.981}^{BMI}\times{BUN}^{-0.069}\times{1.004}^{ALB}\times{0.991}^{UA}\times{1.017}^{HGB}$ |
|  |  | > 0.8 | $129{\times(\frac{Scr}{0.7})}^{-0.360}{\times(\frac{Scys}{0.8})}^{-0.358}\times{0.994}^{Age}$ | | $188{\times(\frac{Scr}{0.7})}^{-0.348}{\times(\frac{Scys}{0.8})}^{-0.261}\times{0.995}^{Age}\times{0.981}^{BMI}\times{BUN}^{-0.069}\times{1.004}^{ALB}\times{0.991}^{UA}\times{1.017}^{HGB}$ |
| Male | ≤ 0.6 | ≤ 0.8 | $135{\times(\frac{Scr}{0.6})}^{-1.193}{\times(\frac{Scys}{0.8})}^{0.033}\times{0.994}^{Age}$ | | $136{\times(\frac{Scr}{0.6})}^{-1.022}{\times(\frac{Scys}{0.8})}^{0.051}\times{0.994}^{Age}\times{0.986}^{BMI}\times{BUN}^{0.050}\times{0.986}^{ALB}\times{0.986}^{UA}\times{1.024}^{HGB}$ |
|  |  | > 0.8 | $135{\times(\frac{Scr}{0.6})}^{-1.193}{\times(\frac{Scys}{0.8})}^{-0.297}\times{0.994}^{Age}$ | | $136{\times(\frac{Scr}{0.6})}^{-1.022}{\times(\frac{Scys}{0.8})}^{-0.252}\times{0.994}^{Age}\times{0.986}^{BMI}\times{BUN}^{0.050}\times{0.986}^{ALB}\times{0.986}^{UA}\times{1.024}^{HGB}$ |
| Male | > 0.6 | ≤ 0.8 | $135{\times(\frac{Scr}{0.6})}^{-0.386}{\times(\frac{Scys}{0.8})}^{0.033}\times{0.994}^{Age}$ | | $136{\times(\frac{Scr}{0.6})}^{-0.394}{\times(\frac{Scys}{0.8})}^{0.051}\times{0.994}^{Age}\times{0.986}^{BMI}\times{BUN}^{0.050}\times{0.986}^{ALB}\times{0.986}^{UA}\times{1.024}^{HGB}$ |
|  |  | > 0.8 | $135{\times(\frac{Scr}{0.6})}^{-0.386}{\times(\frac{Scys}{0.8})}^{-0.297}\times{0.994}^{Age}$ | | $136{\times(\frac{Scr}{0.6})}^{-0.394}{\times(\frac{Scys}{0.8})}^{-0.252}\times{0.994}^{Age}\times{0.986}^{BMI}\times{BUN}^{0.050}\times{0.986}^{ALB}\times{0.986}^{UA}\times{1.024}^{HGB}$ |

**Abbreviations:** CKD-EPI, Chronic Kidney Disease Epidemiology Collaboration; Scr, serum creatinine; Scys, serum cystatin C; BMI, body mass index; BUN, Blood urea nitrogen; ALB, Albumin; UA, Uric acid; HGB, Hemoglobin.

**Additional Table S4. Comparison of the performance of revised 4-variable and 9-variable CKD-EPI equations and 9-variable ANN model: internal validation**

|  | Overall | P value |
| --- | --- | --- |
| Bias – median difference (95% CI) |  |  |
| 4-variable CKD-EPI equation | -0.57 (-2.00 to 0.41) |  |
| 9-variable CKD-EPI equation | -1.98 (-3.27 to -1.06) | 0.06 |
| 9-variable ANN | -0.14 (-1.08 to 0.93) | 0.5 |
|  |  |  |
| Precision – IQR of the difference (95% CI) |  |  |
| 4-variable CKD-EPI equation | 20.66 (18.90 to 22.15) |  |
| 9-variable CKD-EPI equation | 19.18 (17.75 to 20.93) | 0.01 |
| 9-variable ANN | 18.24 (16.96 to 19.55) | < 0.001 |
|  |  |  |
| Accuracy - P30, % (95% CI) |  |  |
| 4-variable CKD-EPI equation | 80.0 (77.5 to 82.6) |  |
| 9-variable CKD-EPI equation | 81.5 (78.9 to 83.8) | 0.1 |
| 9-variable ANN | 83.5 (81.0 to 85.8) | < 0.001 |

**Abbreviations:** GFR, glomerular filtration rate; CKD-EPI, Chronic Kidney Disease Epidemiology Collaboration; ANN, artificial neural network; IQR, interquartile range; CI, confidence interval

| 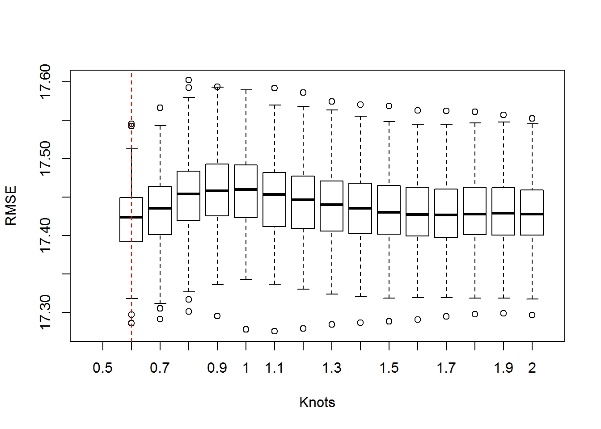  A1. Scr knot for male group | 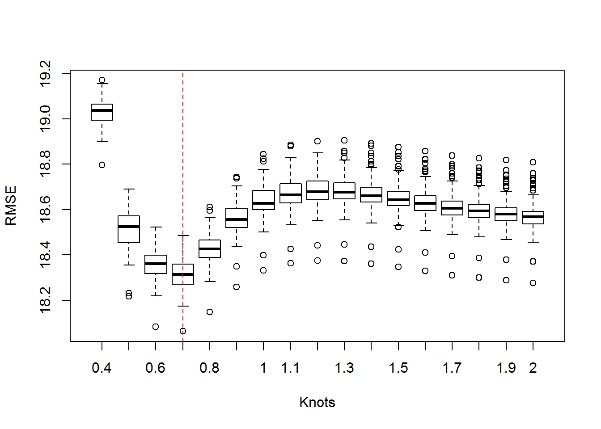  A2. Scr knot for female group |
| --- | --- |
| 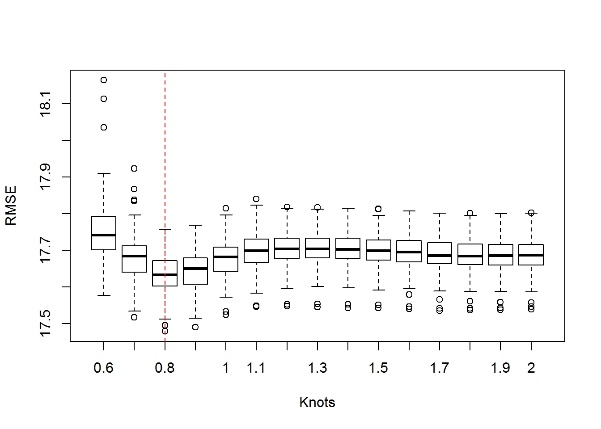  B1. Scys knot for male group | 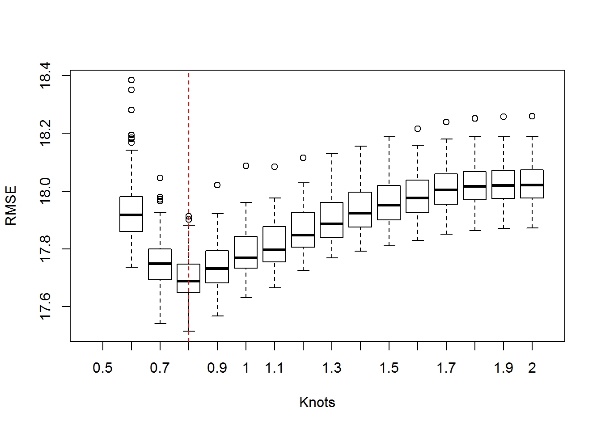  B2. Scys knot for female group |
| **Additional Figure S2**. Box plot of RMSE of 5-fold cross-validation with 100 repetitions for revised CKD-EPI equations. The red vertical dashed line represented the selected knot of Scr and Scys with the lowest average of RMSE over 100 results.  **Abbreviations:** RMSE, root of mean squared error; CKD-EPI, Chronic Kidney Disease Epidemiology Collaboration; Scr, serum creatinine; Scys, serum cystatin C. | |
